# Thirteen Years of Hyoid Suspension Experience in Multilevel OSAHS Surgery: The Short-Term Results of a Bicentric Study

**DOI:** 10.1155/2013/263043

**Published:** 2013-02-20

**Authors:** Pietro Canzi, Anna Berardi, Carmine Tinelli, Filippo Montevecchi, Fabio Pagella, Claudio Vicini, Marco Benazzo

**Affiliations:** ^1^Department of Otorhinolaryngology, University of Pavia and IRCCS Policlinico San Matteo Foundation, Viale Camillo Golgi 19, 27100 Pavia, Italy; ^2^Biometrics Unit, University of Pavia and IRCCS Policlinico San Matteo Foundation, Viale Camillo Golgi 19, 27100 Pavia, Italy; ^3^ENT Unit, Department of Special Surgery, Morgagni-Pierantoni Hospital, Via Forlanini 34, 47121 Forlì, Italy

## Abstract

*Aims*. To evaluate thirteen years of hyoid suspension experience in multilevel OSAHS surgery, for which hyoidthyroidpexia represented the exclusive hypopharyngeal approach applied. *Materials and Methods*. From 1998 to 2011, a bicentric retrospective study was conducted: all adult patients with a diagnosis of OSAHS were enrolled. Specific eligible criteria were established. Pre-/postoperative data concerning ENT and sleep findings were recorded. Recruited subjects were surveilled for a follow-up range from 6 to 18 months. *Results*. A total of 590 hyoid suspensions were evaluated, but only 140 patients met the specific inclusion criteria. A success rate of 67% was obtained. No intraoperative adverse events or major complications occurred. Excessive daytime sleepiness was observed in 28% of nonresponders. Despite the homogeneous candidate anatomy, ENT awake findings changed differently after surgery. Statistical analysis revealed multilevel surgery to be more effective when AHI < 30. Postoperative AHI was statistically not influenced by preoperative BMI. *Conclusions*. Hyoid suspension in multilevel treatment is effective when short-term results are considered. The necessity of a more valuable anatomic-based diagnostic approach is crucial to guide the patient selection. Long-term followups and randomized prospective trials with case-control series are needed to increase the level of evidence of this surgery.

## 1. Introduction

Sleep disordered breathing (SDB) surgery has taken its initial steps from the first tracheotomy [1] up to the pioneering applications of robotics in the new millennium [2]. When Sher and colleagues published the unsatisfying results of surgery in patients with hypopharyngeal obstruction, they were probably still not aware that a great number of hypopharyngeal procedures would be developed [3]. Despite the several techniques reported in the literature (e.g., surgical reduction of the tongue base, tongue base stabilization, genioglossus advancement, mortised genioplasty, tongue radiofrequency treatment, hyoepiglottoplasty, and hyoid suspension), many of them should be critically analyzed anyway because they are extremely invasive. The idea of restoring the retrolingual space acting on the hyoid bone was codified by Riley et al. in 1986 [4], but a few years before experimental attempts in animal models had already been demonstrated [5, 6]. Since then, hyoid suspension has been adopted by many authors finally turning into a stepping stone in SDB surgical management [7]. 

We conducted a bicentric retrospective study to evaluate thirteen years of hyoid suspension experience in multilevel surgery, for which hyoidthyroidpexia represented the exclusive hypopharyngeal approach applied.

## 2. Materials and Methods

### 2.1. Study Design and Patient Selection

From 1998 to 2011, a bicentric retrospective study was conducted at the Otorhinolaryngology Department of the University of Pavia and the Otorhinolaryngology Unit of the Morgagni-Pierantoni Hospital of Forlì. All adult patients (over 18 years of age) with a diagnosis of obstructive sleep apnea hypopnea syndrome (OSAHS) and no other SDB affection were enrolled. This happened after they had accomplished a 6-month or longer lasting CPAP treatment and only after they refused it, in accordance with Mickelson's principles [8]. Eligible criteria also included a multilevel pharyngeal obstruction, for which hyoid suspension represented the exclusive hypopharyngeal surgical approach applied. Institutional review board informed consent was preoperatively obtained from all participants. Recruited subjects were surveilled for a follow-up range from 6 to 18 months.

### 2.2. Clinical and Diagnostic Assessment

A sleep medical diagnosis was executed using a nocturnal, complete, and fully attended polysomnography according to the Associazione Italiana di Medicina del Sonno (Italian Association of Sleep Medicine) Guidelines [9]. OSAHS evidence and CPAP therapy were defined by sleep medicine specialists. Daytime sleepiness was measured using the Epworth Sleepiness Scale (ESS). Weight and height were recorded, and the body mass index (BMI) was calculated. Otorhinolaryngoiatric examination consisted of complete traditional ENT evaluation, upper airway endoscopy using Müller's manoeuvre with the patient in supine position, X-ray cephalometry, and drug-induced sleep endoscopy as previously reported [10]. Patients were staged following the “Nose Oropharynx Hypopharynx” (NOH) classification system introduced, in clinical practice, by the authors since 1999 [11, 12]. Grade and patterns of upper airways collapse were evaluated at the nasal cavities (nose = N), retropalatal space (oropharynx = O), and hypopharyngeal region (hypopharynx = H). The minimal sectional area during Müller's manoeuvre was staged in the following 4 obstructing grades:

 Grade I: <25% collapse;
 Grade II: between 25% and 50% collapse;
 Grade III: between 51% and 75% collapse;
 Grade IV: >75% collapse.

Identification of the obstructing pattern—patient in a supine position—was evaluated according to the shape of the dynamic collapse: anterior-posterior (ap), concentric (c), and transversal (t).

### 2.3. Notes on Hyoid Suspension Technique

The hyoid suspension type II (hyoidthyroidpexia) according to Riley et al.'s modification of 1994 [13] was carried out by the same surgical team (M. Benazzo, F. Pagella, F. Montevecchi, and C. Vicini) on all patients. Key steps in hypopharyngeal reconstruction were the following.

- Anti-Trendelenburg position with neck hyperextension.
- Natural skin crease incision between the hyoid inferior body and the thyroid notch.
- Median strap muscle dissection between two imaginary parasagittal planes crossing the lesser cornu of the hyoid bone.
- Hyoid bone mobilizing test in anteroinferior direction.
- Exposure of the thyroid cartilage (thyroid notch and superior border of thyroid lamina).
- Four stitches (2 on each side) between the hyoid bone and the thyroid notch with reabsorbable sutures (after having reduced neck hyperextension).
- Carry out permanent hyoid fixation after having tested the correct position of the thyroid cartilage below the hyoid bone, following fixed steps which are as follows:
  - double stitching of the anterior parts—hyoid bone, thyroid cartilage—simultaneously,
  - double stitching of the lateral parts—hyoid bone, thyroid cartilage—simultaneously,
  - to achieve an optimal result, the tension of the lateral stitches should be lower than the tension of the anterior ones, in order to avoid hyoid bone fractures.

### 2.4. Postoperative Followup

As part of the standard postoperative protocol, a nasopharyngolaryngoscopic assessment by Müller's manoeuvre was carried out and another polysomnography was performed in all patients from six months onwards after surgery. The apnea hypopnea index (AHI), ESS, NOH, and BMI were assessed during the follow-up period. Responder patients were defined by AHI postoperative values <10 ev/h (recovered) or between 10 ev/h and 20 ev/h (cardiovascular prevention). Nonresponder group was composed of postoperative AHI > 20 ev/h but inferior to preoperative AHI (improved), postoperative AHI = preoperative AHI (unchanged), and postoperative AHI > preoperative AHI (worsened). 

### 2.5. Statistical Analysis

The Shapiro-Wilk test was used to test the normal distribution of quantitative variable. If they were normally distributed, mean and standard deviation were used to summarize them and Student's  *t*-test for dependent samples was used for comparisons between preoperative and postoperative values. If data were not distributed normally, we used median and interquartile range (IQR; 25° and 75° percentile), and nonparametric tests were used to compare data. The  *χ* ^2^  test or Fisher's exact test, as appropriate, was used to determine whether observed differences in proportions between study groups were statistically significant. *P* < 0.05 was considered statistically significant. All tests were two-sided. Statistica 6.0 (StatSoft, Inc., 2006, Tulsa, OK, USA) was used for statistical computations.

## 3. Results

A total of 590 hyoid suspensions were evaluated, but only 140 patients (128 men and 12 women) aged 32–76 years (mean 50.7, median 51) met the specific inclusion criteria. Analyzed data were not distributed normally; therefore, median and interquartile ranges were adopted. Sleep endoscopy findings could not be incorporated in our review due to the lack of a statistical significance in the included population. 

None of the patients had a reduction in the posterior airway space or changes in skeletal measurements at the lateral cephalometric radiograph performed preoperatively. 

We always adopted a single-staged multilevel surgery. Among 140 hyoidthyroidpexia procedures, hyoid suspension was combined as follows:

- nasal + palatal + hyoid surgery = 117 cases,
- nasal + hyoid surgery = 2 cases,
- palatal + hyoid surgery = 21 cases.

Rhinosurgical procedures were carried out in 119 subjects (septoplasty ± turbinoplasty) and 138 patients needed a palatal approach (128 uvulopalatopharyngoplasties with 80 tonsillectomies, 8 laser assisted uvulopalatoplasties, and 2 uvulopalatal flaps). Twenty-three patients previously underwent surgery elsewhere (21 nasal surgery and 2 palatal surgery). No intraoperative adverse events occurred. Major complications were not reported. Two cases of neck seroma, one hyoid bone fracture and one keloid scar development, were noticed without any further significant effects. 

Detailed pre-/postoperative data are abstracted in Table 1.

**Figure 1:**
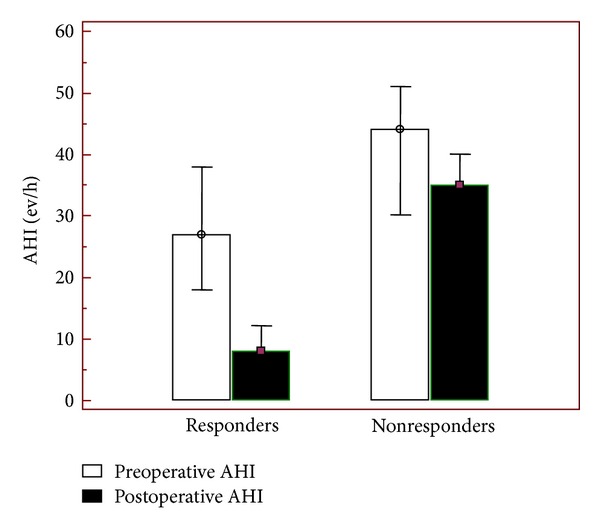
Comparison of pre-/postoperative AHI reduction between responder and nonresponder groups.

Before surgery, 15% of the patients had mild OSAHS (AHI = 5–15 ev/h), 33% moderate (AHI = 16–30 ev/h), and 52% severe (AHI > 30 ev/h), according to the American Sleep Association [14]. Overall, OSAHS level decreased after surgery from a median preoperative AHI value of 31 ev/h (IQR 21 ev/h–44 ev/h) to a median postoperative AHI value of 12 ev/h (IQR 6 ev/h–25 ev/h) with a difference (gain) of  −19 ev/h (*P* < 0.05). The responses to multilevel treatment are summarized in Table 2, indicating a success rate of 67% (*P* < 0.05). Comparison of pre-/postoperative AHI reduction between responder and non-responder groups is shown in Figure 1.

**Figure 2:**
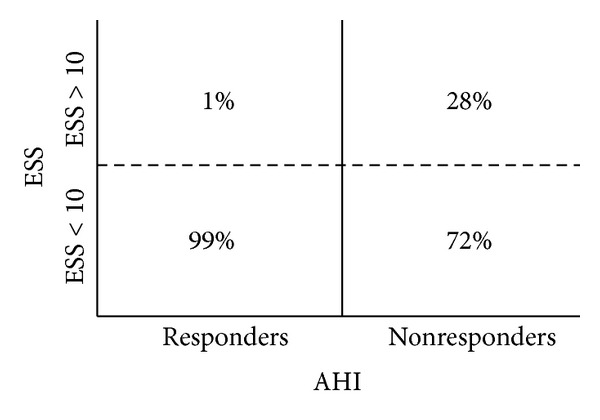
Comparison between postoperative ESS and postoperative AHI.

Excessive daytime sleepiness, also defined as pathological ESS (ESS > 10), characterized the untreated population in 66% of the cases. Hypersomnolence improved significantly after hyoid suspension from a median preoperative ESS of 10 (IQR 8–12) to a median postoperative ESS of 8 (IQR 6–9; gain −2; *P* < 0.05). ESS > 10 was observed in 10% of the postoperative population. The non-responder group included 28% of pathological ESS (Figure 2).

**Figure 3:**
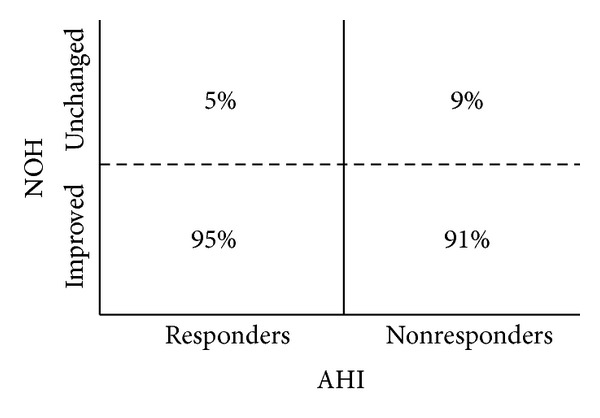
Comparison between postoperative NOH and postoperative AHI.

According to the NOH staging system, the median preoperative finding was N3O4cH3t (IQR N2O3cH2t-N3O4cH3t), thus underlining a homogeneous population with multilevel nose, palate, and hypopharyngeal collapse. The identified pattern of obstruction was typically concentric and transversal in the retropalatal and hypopharyngeal region, respectively. The median postoperative NOH score was N0O1cH1t (IQR N0O0H0-N0O1cH1t; gain −N3O3cH2t; *P* < 0.05) with only 6% (8 patients) of the 140 patients showing no NOH changes postoperatively and 9% of non-responders (4 patients) showing no NOH changes (Figure 3).

**Figure 4:**
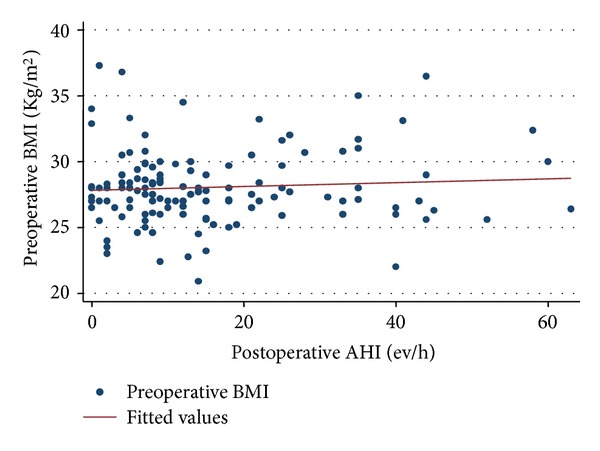
Statistical correlation between preoperative BMI and postoperative AHI.

The BMI outcome did not change after surgery (difference between pre-/postoperative BMI = 0; *P* > 0.05), and post-operative AHI was statistically not influenced by preoperative BMI (Figure 4). Median followup lasted 9 months (IQR 7 months–14 months).

## 4. Discussion

Many questions still remain unanswered concerning the SDB physiopathology, but currently it is generally accepted that pharyngeal airflow obstruction is the consequence of a complex interaction between anatomical and functional factors. Because there is more than one variable involved, it is difficult to define a precise and direct relationship between surgery (cause) and clinical effectiveness (effect). Actually, in order to achieve clinical effectiveness, surgery needs to work on the structural features; in other words, it must act reconstructing the airway wall anatomy. Anatomy is not only different among apneic and nonapneic patients, but it also shows a high individual variability in the OSAHS population. That is why accurate knowledge of the structural defects is crucial in allowing the design of a surgery exactly tailored to each patient [15]. To do this, it is first of all necessary to understand whether a patient is suitable for surgery or not. The selection criteria are, indeed, essential key points to guide surgical treatment decision making. Historically, Fujita developed a specific classification of the airway collapse levels and more recently, Friedman's staging was introduced to increase the surgical response [16, 17]. In 1996 Sher et al. highlighted the hypopharyngeal role, reporting a success rate of 5.3% in all patients belonging to type II-III Fujita's system [3]. Since then, many surgical procedures have developed in order to reshape the hypopharyngeal region.

The hyoid is a u-shaped bone located in the anterior neck midline, at the centre of three force vectors directed, respectively, towards the mandible, sternum, and mastoid process. It gives insertion to the middle constrictor muscles, which form the lateral wall of the hypopharynx. The suspension of this bone to the thyroid cartilage restores the transversal collapse caused by the decreased tone of the middle constrictor muscles in OSAHS patients [13, 18]. Currently, only three clinical trials have been written to investigate the effect of an isolated hyoid suspension for OSAHS, which have reported a response rate ranging from 40% to 53% [13, 19, 20]. A recent MEDLINE meta-analysis proved hyoid suspension to be better recommended in the context of a multilevel surgery in OSAHS patients with retrolingual and hypopharyngeal obstruction (grade of recommendation B) [21]. When genioglossus advancement is associated with hyoid suspension, the success rate reported is apparently not higher than the one after hyoid suspension as the sole procedure to treat hypopharyngeal collapse [22]. Despite the increasing number of reports about hyoidthyroidpexia, there is still insufficient evidence to recommend one suspension technique over another [23]. Nevertheless, as far as we could experience and in accordance with the literature, hyoidthyroidpexia has been shown to be a safe procedure without any major complications [24].

During thirteen years of SDB surgical experience, 590 patients were submitted to hyoid suspension but only 140 of them were evaluated. Many factors caused the numeric reduction of our study which are as follows:

- the limited selection criteria,
- the statistical need of a homogeneous population,
- the relatively recent introduction in our hospitals of a patient data recording system enables to prevent the loss of information,
- lost patients at followup or dropouts.

To the best of our knowledge, we documented the largest cohort of patients submitted to a multilevel surgery, for which hyoidthyroidpexia represents the exclusive hypopharyngeal approach applied. Our success rate of 67% cannot be directly compared with the results reported in the literature because the authors' modalities are different when analyzing response to surgery. In 2006, Kezirian and Goldberg published an evidence-based medicine review collecting all hyoid suspension results and defined as the main surgical goal an AHI reduction of 50% or more and an AHI of less than 20 ev/h [22]. The studies taken into consideration showed a success rate ranging from 17% to 78% of the cases. Borrowing Sher et al.'s criteria [3], we obtained 59% of success rate (*P* < 0.05), in accordance with the results published in the literature. Following our own experience, over and above an accurate identification of the obstructing sites, the successful outcome benefits also from a preoperative AHI. Comparison of pre-/postoperative AHI reduction between responders and non-responders revealed multilevel surgery to be more effective when AHI scored <30 ev/h (*P* < 0.05) (Figure 1). 

Despite the homogeneous anatomy of the enrolled candidates, NOH findings changed differently after surgery as shown in Figure 3. Our results support the necessity of a more valuable anatomic-based diagnostic approach, crucial to guiding surgical treatment decision making. Concerning this matter, drug-induced sleep endoscopy could represent a useful additional instrument in detecting any occult obstruction site, not only during the preoperative assessment but also during the analysis of treatment failures, in order to establish a further salvage therapy [25]. Treatment of the non-responders becomes even harder when clinical aspects are taken into account (Figure 2). The subjective ESS improvement in 72% of the non-responders is surprising and underlines the difficulties in persuading these subjects to continue a therapeutic plan.

There was no functional correlation between BMI observed before surgery and postoperative AHI, that appeared independent, due to the lack of statistically significant percentage of obese patients (BMI > 30) among the preoperative subjects.

Although this is the widest cohort study ever published, it is not free from certain limitations. The absence of a long-term followup and of a randomized prospective design with a case-control series has weakened the strength of our work. The relatively recent introduction of drug-induced sleep endoscopy in our ENT diagnostic equipment did not allow a statistically significant amount of sleep findings in the included population. The awake condition may differ, indeed, quite dramatically from the sleep-breathing one, leading to inaccurate information and limiting the ability to predict upper airway changes during sleep. The adoption of a more complete clinical classification able to record all the patients' features (e.g., NOHL system) could overcome this methodological bias and allow a better patients' selection [12]. 

## 5. Conclusions

Hyoid suspension in multilevel treatment is effective when short-term results are considered. The necessity of a more valuable anatomic-based diagnostic approach is crucial to guide the patient selection. Long-term follow-ups and randomized prospective trials with case-control series are needed to increase the level of evidence of this surgery.

## Figures and Tables

**Table 1:** Pre-/postoperative statistical data.

	Median		Lower quartile		Upper quartile		Gain
	Preoperative	Postoperative	Preoperative	Postoperative	Preoperative	Postoperative	
AHI (ev/h)	31	12	21	6	44	25	−19 (*P* < 0.05)
ESS	10	8	8	6	12	9	−2 (*P* < 0.05)
NOH	N3O4cH3t	N0O1cH1t	N2O3cH2t	N0O0H0	N3O4cH3t	N0O1cH1t	−N3O3cH2t (*P* < 0.05)
BMI (Kg/m^2^)	27.8	27.5	26.5	26.0	29.4	29.0	0 (*P* > 0.05)
Followup (mo)	9 mo		7 mo		14 mo		/

Gain: median postoperative value − median preoperative value. mo: months.

**Table 2:** Multilevel treatment response rates.

	Responders		Nonresponders		
	Recovered (postoperative AHI < 10)	Cardiovascular prevention (10 < postoperative AHI < 20)	Improved (postoperative AHI < preoperative AHI, but postoperative AHI > 20)	Unchanged (postoperative AHI = preoperative AHI)	Worsened (postoperative AHI > preoperative AHI)
Percentage	44%	23%	18%	7%	8%

Total	67%		33%		
